# Quercetin Potentiates Doxorubicin Mediated Antitumor Effects against Liver Cancer through p53/Bcl-xl

**DOI:** 10.1371/journal.pone.0051764

**Published:** 2012-12-11

**Authors:** Guanyu Wang, Jiawei Zhang, Luying Liu, Sherven Sharma, Qinghua Dong

**Affiliations:** 1 Department of General Surgery, Sir Run Run Shaw Hospital, School of Medicine, Zhejiang University, Hangzhou, Zhejiang, China; 2 Cancer Institute, The Second Affiliated Hospital, Zhejiang University, Hangzhou, Zhejiang, China; 3 Department of Radiotherapy, Zhejiang Cancer Hospital, Hangzhou, Zhejiang, China; 4 David Geffen School of Medicine at UCLA, and the Veterans Affairs, Los Angeles, California, United States of America; 5 Biomedical Research Center, Sir Run Run Shaw Hospital, School of Medicine, Zhejiang University, Hangzhou, Zhejiang, China; 6 Key Laboratory of Cancer Prevention and Intervention, China National Ministry of Education, Key Laboratory of Cancer Biotherapy of Zhejiang Province, Hangzhou, China; Ain Shams University, Egypt

## Abstract

**Background:**

The dose-dependent toxicities of doxorubicin (DOX) limit its clinical applications, particularly in drug-resistant cancers, such as liver cancer. In this study, we investigated the role of quercetin on the antitumor effects of DOX on liver cancer cells and its ability to provide protection against DOX-mediated liver damage in mice.

**Methodology and Results:**

The MTT and Annexin V/PI staining assay demonstrated that quercetin selectively sensitized DOX-induced cytotoxicity against liver cancer cells while protecting normal liver cells. The increase in DOX-mediated apoptosis in hepatoma cells by quercetin was p53-dependent and occurred by downregulating Bcl-xl expression. Z-VAD-fmk (caspase inhibitor), pifithrin-α (p53 inhibitor), or overexpressed Bcl-xl decreased the effects of quercetin on DOX-mediated apoptosis. The combined treatment of quercetin and DOX significantly reduced the growth of liver cancer xenografts in mice. Moreover, quercetin decreased the serum levels of alanine aminotransferase and aspartate aminotransferase that were increased in DOX-treated mice. Quercetin also reversed the DOX-induced pathological changes in mice livers.

**Conclusion and Significance:**

These results indicate that quercetin potentiated the antitumor effects of DOX on liver cancer cells while protecting normal liver cells. Therefore, the development of quercetin may be beneficial in a combined treatment with DOX for increased therapeutic efficacy against liver cancer.

## Introduction

Hepatocellular carcinoma (HCC) is one of the most common types of liver cancer and the fourth leading cause of cancer deaths worldwide [1]. The lack of biomarkers that detect surgically resectable early stage of a disease has caused the manifestation of advanced stages in most patients when surgical resection is no longer feasible [2]. Therefore, chemotherapy remains the viable option for the treatment of inoperable HCC patients.

Over the years, doxorubicin (DOX) has become a routinely and widely used agent in HCC treatment. However, studies have shown that some cancer cells, including hepatoma, are resistant to the apoptotic effects of DOX [3]. Furthermore, DOX-based chemotherapy is associated with severe side effects to non-tumorous tissues, such as the heart, liver, and kidney, greatly limiting its clinical applications [4], [5]. Benjamin described eight patients with impaired liver function who developed severe pancytopenia and mucositis while receiving DOX, prompting experts to reduce the dosage because of altered hepatic function [6]. Thus, improved therapeutic regimens that potentiate DOX effects, which allow dose reduction and protection of non-tumorous tissues, are needed to improve the treatment of liver cancer patients.

The mechanisms of DOX-mediated cytotoxicity in cancer cells and normal tissues are different [7]. DOX toxicity in cancer cells primarily occurs through DNA intercalation and damage [8], whereas DOX-induced cardiotoxicity or hepatotoxicity mainly occurs by generating oxygen free radicals, which is inhibited by free radical scavengers [9]. This difference in DOX-mediated toxicity in cancer and normal cells can be investigated to improve the antitumor effects of DOX with combinatorial approaches that allow the dose reduction of DOX while protecting normal cells.

Quercetin (3, 3′, 4′, 5, 7-pentahydroxyflavone), an important dietary flavonoid present in several fruits and vegetables, exhibits antioxidant, anti-inflammatory, and anticancer properties [10]. Quercetin has received increasing attention as a pro-apoptotic flavonoid with specific and almost exclusive activity on tumor cells rather than normal, non-transformed cells [11], [12]. However, the mechanisms by which quercetin exerts its anti-proliferative and apoptotic activities remain unclear. Several *in vitro* and *in vivo* studies have evaluated quercetin combined with DOX for breast cancer and leukemia treatments, revealing synergistic effects [13]–[15]. In murine breast cancer models, a combination of dietary quercetin and intratumoral injection of DOX reduces the tumor volume and metastatic spread [13]. Although quercetin reverses DOX-induced multidrug resistance in human myelogenous leukemia cells [14], [16], no studies on the efficacy of quercetin with DOX against liver cancer have been reported yet.

Antioxidants have beneficial effects against DOX-induced toxicity in mice and rats [17]. Quercetin has a protective effect against DOX-induced cardiotoxicity in mice, but the mechanisms remain unclear [18]. Quercetin also exhibits a protective effect against AFB1-mediated liver damage *in vivo* by promoting antioxidative defense systems and inhibiting lipid peroxidation [19]. Furthermore, quercetin reduces the hepatic cytochrome P450 content and increases the hepatic glutathione S-transferase (GST) activity involved in the activation/detoxification of chemical mutagens/carcinogens [20]–[22]. These findings suggest that quercetin may provide protection against DOX-mediated liver damage.

The present study aims to evaluate the effects of the combination of DOX chemotherapeutic agent with the natural compound quercetin on human liver cancer and normal cells. The potential function of quercetin as a liver protective agent during DOX treatment in mice was also examined.

Our results demonstrate that the quercetin-enhanced DOX-mediated apoptosis in hepatoma cells is p53-dependent and occurs by downregulating Bcl-xl. Moreover, quercetin exhibits a protective effect against DOX-induced hepatotoxicity in mice.

## Results

### Quercetin selectively potentiates DOX cytotoxicity in liver cancer cells but not in normal liver cells

**Figure 1:**
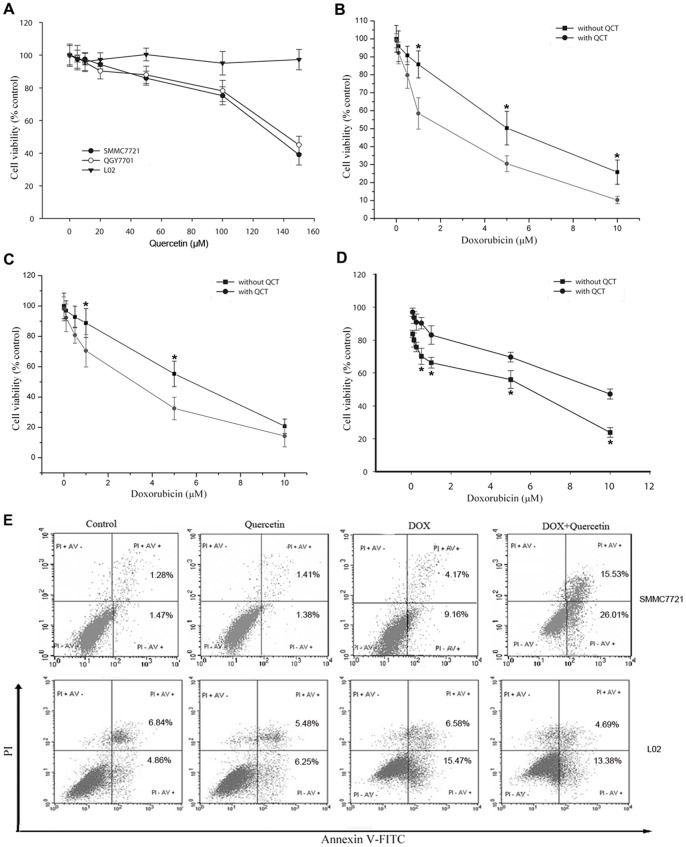
Quercetin potentiates the effect of DOX on proliferation and apoptosis in liver cancer cells, but not in normal liver cells. (A) Effect of quercetin on the proliferation of SMMC7721 and QGY7701 liver cancer cells, and L02 normal liver cells. The cells were incubated with quercetin (0 µM to 150 µM) for 48 h and subjected to an MTT assay to determine the proliferation rate. (B) Quercetin sensitized SMMC7721 cells to DOX. (C) Quercetin sensitized QGY7701 cells to DOX. (D) Quercetin partially reduced the DOX-induced growth inhibition in L02 cells. The cells were incubated with DOX (1 µM) and/or quercetin (20 µM) (B, C, and D) for 24 h, and then subjected to an MTT assay. Data are presented as mean ± S.D. of three independent experiments. (E) SMMC7721 and L02 cells were incubated with DOX (1 µM) and/or quercetin (20 µM) for 24 h, and then analyzed by flow cytometry using Annexin V/PI staining to discriminate the live cells (Annexin V−/PI−), early apoptotic cells (Annexin V+/PI−), necrosis or late apoptotic cells (Annexin V+/PI+), and dead cells (Annexin V−/PI+). *P<0.05 vs. cells co-treated with quercetin.

Cultures were exposed to quercetin at doses of 0 µM to 150 µM to determine the effects of quercetin on liver cancer cells and normal cells. Dimethylsulfoxide (DMSO; 0.1%) was used as the negative control. After 48 h, a dose-dependent reduction in cancer cell viability (IC_50_ value SMMC7721 cells  = 133 µM and QGY7701 cells  = 142 µM) was observed (Fig. 1A). Quercetin did not affect normal liver cell (L02) growth even at high concentrations (>100 µM). Considering that 12 µM of quercetin serum concentration did not induce any associated side-effects on humans and its cytotoxicity on several cancer cell lines [23], [24], we selected 20 µM of quercetin for drug combination studies. Quercetin (20 µM) increased DOX-induced liver cancer cell death and reduced the IC_50_ value of DOX against hepatoma SMMC7721 cells (5.1 µM to 2.2 µM; Fig. 1B) and QGY7701 cells (6.2 µM to 2.7 µM; Fig. 1C). By contrast, pretreatment with 20 µM of quercetin reduced the DOX-mediated cytotoxicity in normal liver L02 cells (Fig. 1D). In human plasma, the peak and the steady-state concentration of DOX are 5 µM and 25 nM to 500 nM, respectively [25]; thus, we selected 1 µM of DOX for the drug combination studies to represent relevant plasma levels in DOX-treated patients. Annexin V/PI staining was performed to investigate whether or not DOX-induced cell death in hepatoma cells occurs through apoptosis. DOX (1 µM) treatment induced apoptosis in 13% of the SMMC7721 cells; the co-treatment with quercetin increased the percentage of apoptotic cells to 42% (Fig. 1E). The percentage of DOX-induced apoptosis alone or the co-treatment with quercetin in L02 cells were approximately 22% and 18%, respectively, suggesting that quercetin did not sensitize the normal liver cells to DOX-induced apoptosis. Similar results were confirmed by Hoechst staining (data not shown). These results suggested a preferential potentiation effect of quercetin on DOX-mediated cytotoxicity in liver cancer cells, but not in normal liver cells.

### Quercetin enhances DOX-induced caspase activation in liver cancer cells

**Figure 2:**
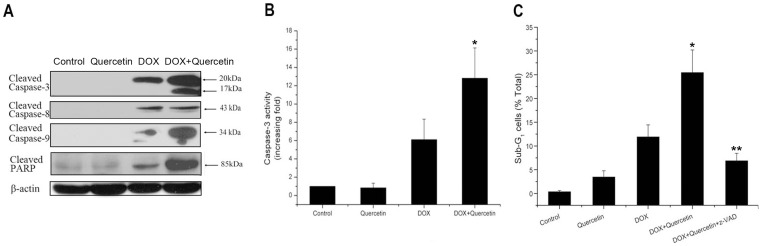
Quercetin enhances DOX-induced caspase activation. (A) Cleaved caspases-3, -8, and -9 and PARP expression were assessed by western blotting in SMMC7721 cells treated with DOX (1 μM) and/or quercetin (20 μM) for 24 hours. β-actin was used as an internal control. (B) Caspase-3 activity assay of DOX- (1 µM) and/or quercetin-(20 µM) treated SMMC7721 cells for 24 h. Cell lysates were incubated with fluorogenic caspase-3 substrate for 1 h at 37°C. Caspase-3 activity was normalized to cell lysate protein and expressed as fold activation compared with the control. *P<0.05 vs. DOX-treated cells. (C) Caspase inhibitor Z-VAD-fmk reduced the effect of quercetin on DOX-induced apoptosis in QGY7701 cells examined by PI staining. Control: 0.5% dimethyl sulfoxide; quercetin: 20 µM; DOX: 1 µM; DOX + quercetin: DOX 1 µM plus quercetin 20 µM; Z-VAD-fmk + DOX + quercetin: cells were pretreated with 25 µM of z-VAD-fmk for 1.5 h and further treated with DOX plus quercetin for 24 h. Data are presented as mean ± S.D. of three independent experiments. *P<0.05 vs. DOX-treated cells. **P<0.01 vs. DOX + quercetin-treated cells.

Caspase activation has an important function in both intrinsic and extrinsic apoptotic pathways. Thus, we also examined the effect of quercetin on DOX-induced changes in caspases. Quercetin did not directly activate caspases, but strongly enhanced the DOX-induced caspase-9 activation, not caspase-8 activation, in SMMC7721 cells (Fig. 2A). Activated caspase-3, the major effector of caspases, and PARP, the main substrate of caspases, were also evaluated. Quercetin alone did not induce any cleavage of caspase-3 in SMMC7721 cells; DOX induced a 20 kDa cleavage formation. However, the co-treatment of quercetin and DOX induced the cleavage of procaspase-3 into a p20 intermediate, which subsequently cleaved to the active p17 subunit. More PARP proteins were cleaved into 85 kDa forms after the combination treatment was administered compared with DOX treatment alone. Similar activation patterns of caspases and cleavage of PARP were observed in QGY7701 cells (data not shown). The caspase-3 activity assay confirmed that quercetin significantly increased the DOX-induced caspase-3 activity in SMMC7721 cells (Fig. 2B). The effects of the broad-spectrum caspase inhibitor Z-VAD-fmk on liver cancer cell apoptosis were determined by PI staining to confirm that the action of quercetin on DOX was caspase dependent. The co-treatment-induced accumulation of sub-G1 phase cell populations in QGY7701 cells was significantly decreased (26% to 7%) by the pretreatment with Z-VAD-fmk (Fig. 2C). The sub-G1 phase cell population in SMMC7721 cells also decreased from 39% to 8% (data not shown). These results indicated that quercetin potentiated DOX-induced apoptosis in liver cancer cells by activating caspases-9 and -3 of the intrinsic pathway.

### Quercetin potentiates DOX-induced apoptosis in liver cancer cells through Bcl-xl/Bax-mediated mitochondrial pathway

**Figure 3:**
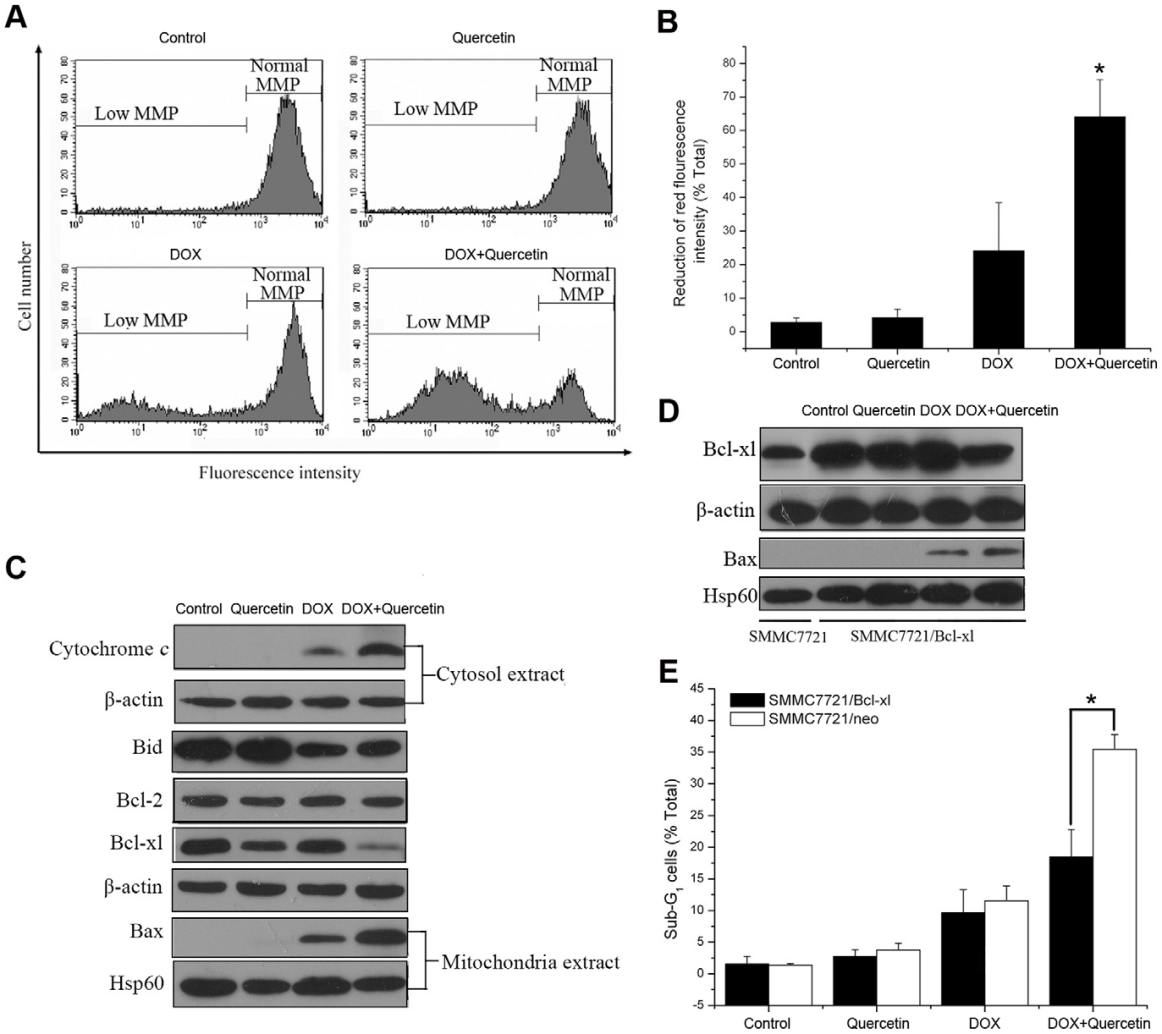
Quercetin potentiates DOX-induced apoptosis in liver cancer cells through Bcl-xl/Bax-mediated mitochondrial pathway. (A, B) Effect of DOX and/or quercetin on the mitochondrial membrane potential breakdown in SMMC7721 cells treated with DOX (1 µM) and/or quercetin (20 µM) for 24 h. JC-1 is observed as green fluorescing monomers in the cytosol or as red fluorescing aggregates in intact mitochondria. The reduction of red fluorescence intensity indicates mitochondrial breakdown with intact membrane potential. *P<0.01 vs. DOX-treated cells. (C) Western blot analysis of the total expression of Bid, Bcl-2, and Bcl-xl, as well as the mitochondrial distribution of Bax and the cytosol distribution of cytochrome *c* in SMMC7721 cells treated with DOX (1 µM) and/or quercetin (20 µM) for 24 h. β-actin was used as an internal control for the total protein and cytosol protein. Hsp60 was used as an internal control for the mitochondrial protein. (D) Western blot analysis of the mitochondrial distribution of Bax and the total expression of Bcl-xl in SMMC7721 cells transfected with Bcl-xl expression vector and treated with DOX (1 µM) and/or quercetin (20 µM) for 24 h. (E) Effect of DOX and/or quercetin on the apoptosis of SMMC7721/Bcl-xl and SMMC7721/neo cells assayed by PI staining after DOX (1 µM) and/or quercetin (20 µM) was administered for 24 h. *P<0.01, SMMC7721/neo vs. SMMC7721/Bcl-xl cells. Data are presented as mean ± S.D. of three independent experiments.

Experiments were conducted using JC-1 to investigate the potential involvement of mitochondrial membrane disruption. Quantitative measurements showed that SMMC7721 cells treated with quercetin alone did not exhibit any change compared with the control. DOX treatment induced a moderate reduction of red fluorescence, indicating a decrease in the mitochondrial membrane potential, whereas the co-treatment of quercetin and DOX induced a sharp, significant reduction (Figs. 3A and B). Consistent with the loss of mitochondrial membrane, the co-treatment of SMMC7721 cells with quercetin and DOX released more cytochrome *c* from the mitochondria to the cytosol compared with that of DOX treatment alone (Fig. 3C).

We examined Bcl-2 family proteins expression level to investigate the potential molecular targets upstream of caspase-9 pathways. Bcl-2 expression level did not change after various treatments were performed. The co-treatment of DOX and quercetin did not affect the DOX-induced decrease in Bid expression level. Surprisingly, the co-treatment caused a sharp reduction in Bcl-xl expression, whereas DOX or quercetin treatment alone did not affect Bcl-xl expression (Fig. 3C). Accordingly, the co-treatment of SMMC7721 cells with quercetin and DOX greatly increased Bax translocation from the cytosol into the mitochondria compared with DOX treatment alone, which only caused a moderate increase in Bax translocation.

SMMC7721 cells were stably transduced using the Bcl-xl expression vector to confirm the important function of Bcl-xl protein in the effects of quercetin on DOX-induced apoptosis. The overexpression of Bcl-xl in SMMC7721 cells significantly inhibited Bax translocation to the mitochondria which was induced by the co-treatment of DOX and quercetin (Fig. 3D), and reduced the potentiation effect of quercetin on DOX-induced apoptosis (Fig. 3E). These results suggested that quercetin potentiates DOX-induced apoptosis in hepatoma cells through the mitochondrial pathway by downregulating Bcl-xl protein and subsequently increasing Bax translocation into the mitochondria.

### Quercetin enhancement of DOX-induced apoptosis in liver cancer cells is p53-dependent

**Figure 4:**
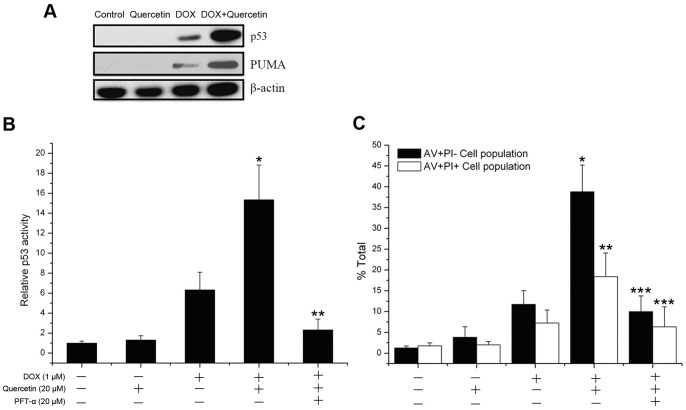
Quercetin potentiates DOX-induced apoptosis of SMMC7721 cells in a p53-dependent manner. (A) p53 and PUMA expressions were assessed by western blot in SMMC7721 cells treated with DOX (1 µM) and/or quercetin (20 µM) for 24 h. β-actin was used as an internal control. (B) The luciferase assay of DOX- and quercetin-induced p53 activation. SMMC7721 cells were pretreated for 1 h with 2 µM of pifithrin-α, and then treated with DOX (1 µM) or quercetin (20 µM) for 12 h. p53 activity was determined by luciferase activity assay. Data are presented as mean ± S.D. of three independent experiments. *P<0.01 vs. DOX-treated cells. **P<0.001 vs. DOX + quercetin-treated cells. (C) SMMC7721 cells were pretreated with 2 µM of pfithrin-α for 1 h, and then treated with DOX (1 µM) and/or quercetin (20 µM) for 24 h. Apoptosis rates were assayed by Annexin V/PI staining. Data are presented as mean ± S.D. of three independent experiments. *P<0.001 vs. DOX-treated cells. **P<0.01 vs. DOX-treated cells. ***P<0.001 vs. DOX + quercetin-treated cells.

Activated p53-mediated promotion of apoptosis in tumor cells is an important mechanism of antitumor drugs, such as DOX [26]. Therefore, we investigated whether p53 protein is involved in the potentiation effect of quercetin on DOX-induced apoptosis in liver cancer cells. No detectable p53 protein expression was observed in the control or quercetin-treated cells, whereas the co-treatment of quercetin and DOX significantly increased the expression level of p53 protein induced by DOX in SMMC7721 cells (Fig. 4A). p53 upregulated modulator of apoptosis (PUMA), a pro-apoptotic BH3-only protein, is a direct transcriptional target of p53. Quercetin subsequently increased the DOX-induced PUMA expression levels. SMMC7721 cells were transiently transfected with p53-luciferase plasmid and exposed to different treatments to clarify the function of p53 in the potentiation effect of quercetin on DOX-induced apoptosis. Quercetin alone did not enhance the p53 activity. By contrast, the combined treatment with DOX and DOX treatment alone significantly enhanced the p53 activity by 15- and 6-fold, respectively, compared with the control group. Moreover, pifithrin-α, a specific p53 inhibitor, did not affect cell survival, but significantly inhibited p53 activity (2.4-fold), which was enhanced by the combined quercetin and DOX treatment (Fig. 4B), and reduced the potentiation effect of quercetin on DOX-induced apoptosis (Fig. 4C). These results suggested that the enhanced p53 activity is necessary to stimulate the potentiation effect of quercetin on DOX-induced apoptosis.

### Quercetin increases DOX–induced growth inhibition of SMMC7721 xenografts in mice

**Figure 5:**
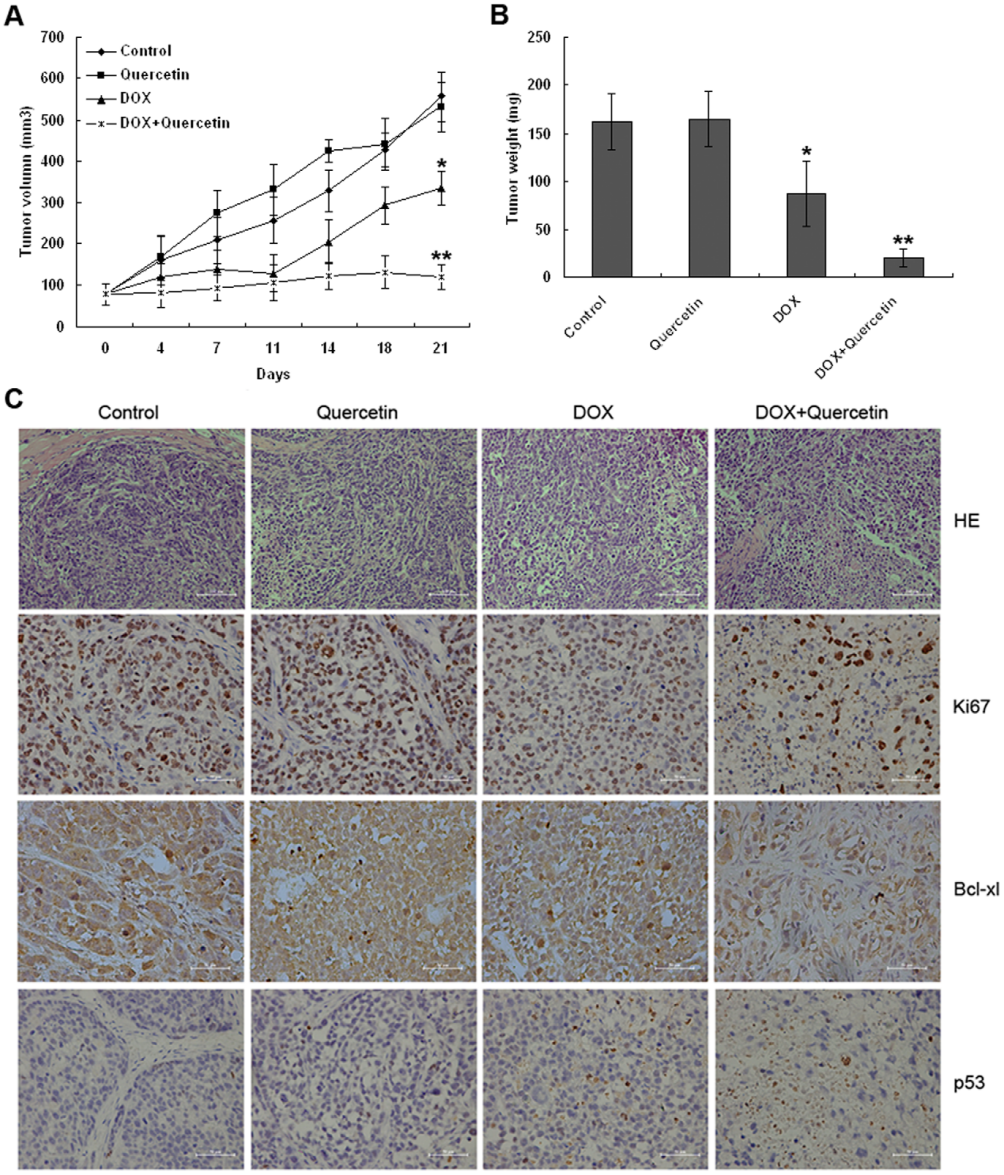
Quercetin potentiates SMMC7721 tumor growth inhibition by DOX *in vivo*. SMMC7721 cell-derived tumors were developed in nude mice and treated with saline, quercetin, DOX, and DOX + quercetin. (A) Tumor growth was monitored by measuring the tumor volume for three weeks. (*n* = 5 mice per group). *P<0.01 vs. the control and quercetin-treated groups. **P<0.01 vs. DOX-treated group. (B) At the end of three weeks, the tumors were collected and weighed. DOX reduced the tumor size compared with the control and quercetin-treated groups (*P<0.05). Co-treatment significantly reduced the tumor size compared with other treatment groups (**P<0.001). (C) Tumor samples were subjected to hematoxylin and eosin staining and immunohistochemical analysis using Ki67, Bcl-xl, and p53 antibodies. Co-treated tumors showed a significant reduction in Ki67 and Bcl-xl expression as well as an increase in p53 expression compared with other treated tumors (*P*<0.05).

The effects of quercetin on DOX in our cell culture model *in vitro* were validated in this study. Hence, we evaluated whether quercetin can potentiate the antitumor effect of DOX in SMMC7721 tumor xenografts in nude mice. SMMC7721 single cell suspensions were injected s.c. into the flanks of six-week-old immune-deficient mice, the mice were then divided into four groups 10 days post tumor inoculation when tumors were palpable: 1) normal saline control group (i.p., 3×/wk); 2) quercetin (100 mg/kg, i.p., 3×/wk); 3) DOX (4 mg/kg, i.p., 1×/wk); and 4) DOX (4 mg/kg, i.p., 1×/wk) plus quercetin (100 mg/kg, i.p., 3×/wk). For the DOX group, treatments were administered until a cumulative dose of 12 mg/kg was reached. Quercetin alone did not inhibit tumor growth; instead, quercetin promoted tumor growth for the first 14 days, and then slowed the tumor growth for the next 7 days compared with the control group. At the end of the experiment, the average tumor volume of quercetin treatment was 531.3 mm^3^, similar to that of the control group. DOX treatment significantly reduced the tumor volumes (334.7 mm^3^) compared with the control group. A significant decrease in the tumor volume of the co-treated xenografts was observed from day four of post-treatment until the end of the experiment (Fig. 5A). After co-treatment was administered, the tumors grew at a reduced rate with the start and final average tumor volumes of 76.5 and 118.1 mm^3^, respectively.

The average tumor weight in the control group reached 163 mg, whereas the quercetin-treated group elicited no effect on SMMC7721 tumor growth. DOX treatment resulted in approximately 50% tumor growth inhibition compared with the control group; the average tumor weight decreased to 87 mg. The co-treatment of DOX and quercetin elicited the most effective tumor growth inhibition and resulted in an average tumor weight of 20 mg (Fig. 5B). The data indicated that quercetin increases the antitumor activity of DOX *in vivo*.

Histologically, the tumors were considerably less cellular and composed primarily of acellular material in the co-treated xenografts. By contrast, the tumor cells from mice that received the vehicle control and quercetin treatment were arranged as nests separated by bundles of extracellular matrix (Fig. 5C). A low number of tumors were found in DOX-treated mice. Tumor sections from nude mice were assessed for Ki67 expression to investigate the effect of the combined treatment on tumor cell proliferation (Fig. 5C). After three weeks of treatment, 24%, 62%, 91% and 92% of Ki67+ tumor cells were found in the co-, DOX-, and vehicle control or quercetin-treated mice, respectively. Additional immunohistochemical analyses were performed to confirm the *in vitro* findings on the mechanisms by which quercetin exhibits potentiation effect with DOX *in vivo*. Quercetin and DOX co-treated tumors exhibitd increased p53 levels and decreased Bcl-xl levels compared with the control group (Fig. 5C), consistent with our *in vitro* findings.

### Quercetin attenuates DOX-induced hepatotoxicity *in vivo*

Considering our *in vitro* observations that quercetin can decrease DOX cytotoxicity in normal liver cells (Fig. 1D), we further investigated its effect on DOX-induced hepatotoxicity *in vivo*. C57BL/6 mice were treated with DOX at 20 mg/kg (i.p.), a dose that causes acute toxicity [27]. The acute hepatotoxicity of DOX was clearly revealed by the increase in serum biochemical markers, particularly alanine aminotransferase (ALT) and aspartate aminotransferase (AST). DOX treatment resulted in a significant increase of 765% and 378% in ALT and AST, respectively, compared with the control group (Table 1). Quercetin supplementation alone did not exhibit significant changes in the biochemical markers. Conversely, the co-treatment of DOX and quercetin resulted in a partial reversal of DOX-induced serum increase in ALT and AST (*p*<0.05).

**Table 1:** Effect of quercetin on DOX-induced liver enzyme indices: alanine aminotransferase (ALT) and aspartate aminotransferase (AST) changes in mice.

Enzyme assays (U/L)	Group I (Control)	Group II (QCT)	Group III (DOX)	Group IV (DOX + QCT)
ALT	32.1±4.1	29.2±5.3	245.5±24.7*	105.3±30.1^**^
AST	190.2±26.5	159.2±21.2	718.5±52.4*	442.5±38.9^**^

Values are expressed as mean ± SD for six animals in each group.

* *P<*0.01 vs. the control and quercetin-treated mice. ^**^ *P<*0.05 vs. DOX-treated mice.

**Figure 6:**
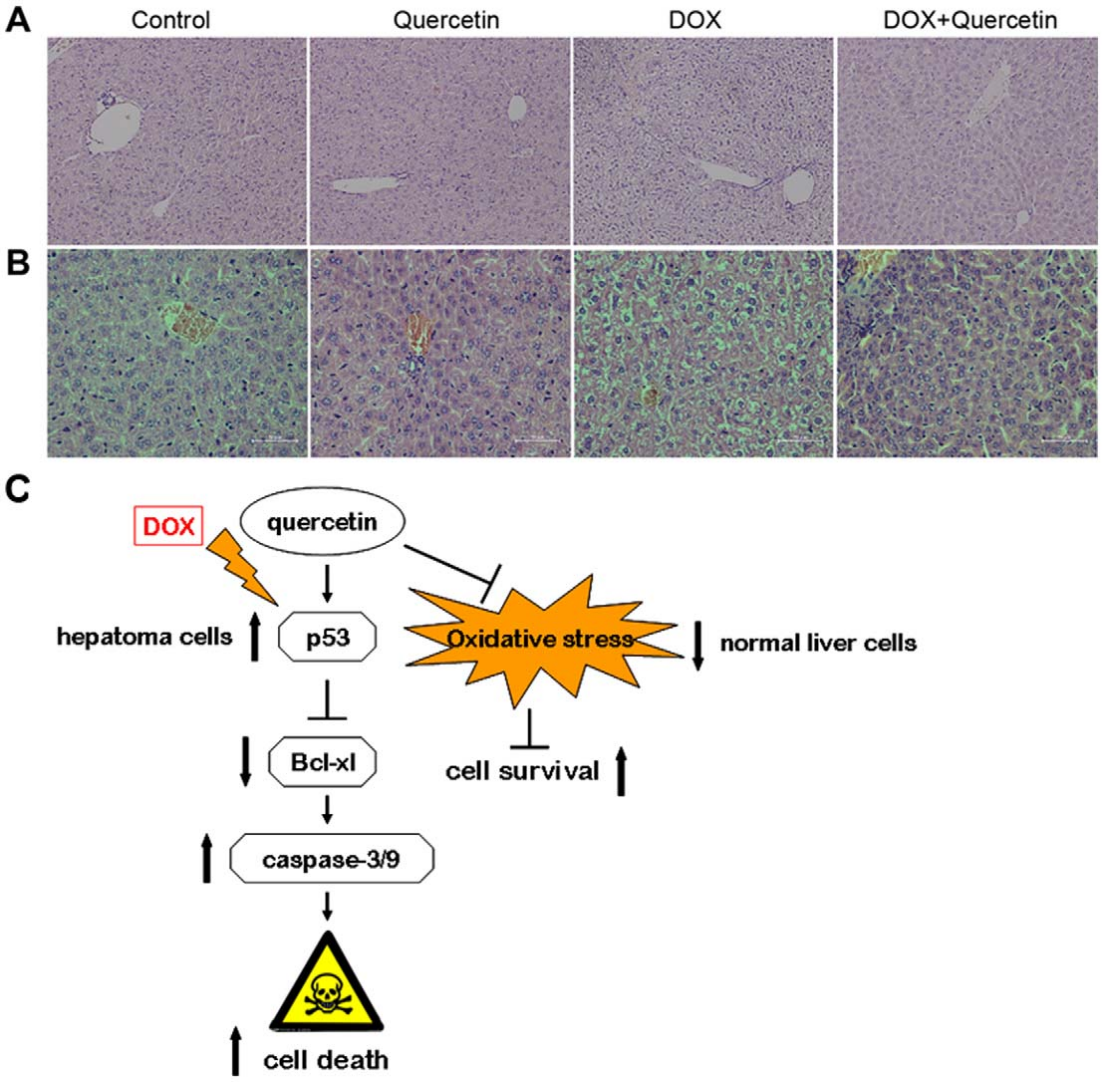
Histological changes in mice livers that received DOX and/or quercetin. (A) C57BL/6 mice were treated with quercetin (100 mg/kg/day, p.o.) four days before the i.p. administration of DOX (20 mg/kg). Livers were removed five days after DOX treatment, and the sections were stained with hematoxylin and eosin (H&E; magnification 200×). (B) SMMC7721 cell-derived tumors were developed in nude mice and treated with saline, quercetin, DOX, and DOX + quercetin. At the end of three weeks, livers were collected and stained with H&E. (C) A model of the effect of quercetion on DOX in liver cells. In this model, quercetin increases DOX-induced p53 expression in liver cancer cells and reduces Bcl-xl expression, thereby increasing caspase −3/−9 activity and potentiating DOX-induced cell death in liver cancer cells. Quercetin decreases DOX-induced oxidative stress and increases cell survival in normal liver cells.

The microscopic examination revealed that the control and quercetin-treated hepatic tissues have large normal polygonal cells with prominent round nuclei and eosinophilic cytoplasm, as well as a few spaced hepatic sinusoids arranged in-between the hepatic cords (Fig. 6A). By contrast, the mice that received 20 mg/kg of DOX showed massive hepatotoxicity, with the dissolution of involved hepatic cords, which appeared as empty vacuoles aligned by strands of necrotic hepatocytes and kupffer cells proliferated in a diffused manner between the fatty degenerated hepatocytes. Histopathological evidence revealed that hepatic damage after DOX treatment was significantly reduced upon co-treatment with quercetin.

We investigated the DOX-induced subchronic hepatic damages in mice using SMMC7721 xenografts. The DOX-treated group received DOX once a week (4 mg/kg, i.p.) until a cumulative dose of 12 mg/kg was reached. The hepatic tissue of the DOX-treated group showed pathological changes similar to those of acute DOX-treated C57BL/6 mice (Fig. 6B). Quercetin reversed the DOX-induced liver damage in nude mice with SMMC7721 tumor xenografts.

## Discussion

Although DOX is a potent anticancer drug, its clinical use is limited by its toxicity to normal tissues, such as the heart and liver. Another limitation of its effectiveness is the development of multidrug resistance by cancer cells. Some flavonoids exhibit increased anti-tumor effects with chemotherapeutic agents, such as quercetin [28]. In *in vitro* and breast cancer animal models, quercetin improves the therapeutic index of DOX in breast cancer cells by interfering with cell metabolism, GST activity, and cytoskeleton. By contrast, quercetin reduces DOX cytotoxicity in non-tumorous mammary cells [13]–[15]. Although the co-treatment of quercetin and DOX may be developed to a new chemotherapeutic combination for breast cancer therapy, whether or not quercetin elicits the same effect on DOX in other types of cancers, in which DOX is used as first-line treatment, such as liver cancer, remains unknown. The current study investigated the efficacy of quercetin with DOX on liver cancer cells and its protective effect against DOX-induced hepatotoxicity in mice.

Several studies have shown that the IC_50_ for quercetin in various tumors ranges from 7 nM to >100 µM. In contrast to other types of human cancer cells, quercetin exhibits anti-proliferative and pro-apoptotic effects at low concentrations [23], such as 21 µM for MDA-MB-468 human breast cancer cells [24]. Several reports and our results demonstrated that human hepatoma cells are considered resistant to quercetin because the IC_50_ value is >100 µM. Human hepatoma cells are also resistant to DOX (IC_50_ value >5 µM) and the peak DOX concentration in human plasma is 5 µM. Our results also revealed that the co-treatment of quercetin (20 µM) and DOX (1 µM) significantly potentiates the antitumor effects of DOX in human liver cancer cells. Cancer cells exhibit their resistance to chemotherapeutic agents by expelling the intracellular anti-cancer drugs out of the cells through P-gp and other drug pumps. Quercetin can inhibit the P-gp pump efflux activity dose-dependently and induce apoptosis in resistant human myeloid leukemia and breast cancer cells [29]–[31]; thus, the facilitated DOX-induced apoptosis maybe the result of quercetin-induced P-gp inhibition, followed by an increase in intracellular DOX, and a consequently enhanced apoptosis [32]. However, low doses of DOX can slightly increase mdr1 mRNA, but not protein expression [33]. For MRP1, no detectable MRP1 mRNA in DOX-treated SMMC7721 cells was found [34]. With confocal microscopic technologies, we observed that the intracellular distribution of DOX in these hepatoma cells was not affected by quercetin (data not shown). Thus, quercetin likely potentiates DOX-induced apoptosis, but not through its action on drug retention and efflux. Further studies are needed to determine whether or not quercetin sensitizes DOX-resistant liver cancer cells by affecting mdr-related gene expression.

DOX-induced apoptosis is associated with two distinct apoptosis pathways: the death receptor pathway by activating caspase-8 and the mitochondrial pathway by activating caspase-9 [35]. The mitochondrial pathway is the major mechanism of DOX-induced apoptosis [36], in which the central process involves the permeabilization of the outer mitochondrial membrane with the subsequent release of several pro-apoptotic factors into the cytosol [37]. Cytochrome *c*, one of the pro-apoptotic factors, CARD adapter protein APAF-1, and pro-caspase-9 assemble in the cytosol to form the apoptosome. Pro-caspase-9 is then autoproteolytically processed and triggers the activation of effector caspases, thereby leading to the cleavage of several substrates. We found that quercetin significantly increased DOX-induced loss of the mitochondrial membrane potential, indicating the increase in mitochondrial breakdown. Substantially enhanced release of cytochrome *c* and cleavage of pro-caspase-9 subsequently occurred, leading to the increase in caspase-3 activity and PARP degradation. Z-VAD-fmk blocked the quercetin-enhanced, DOX-induced apoptosis, suggesting that quercetin sensitizes DOX-induced apoptosis in liver cancer cells mainly through the mitochondrial pathway.

Numerous reports indicated that p53 tumor suppressor protein regulates mitochondria-directed apoptosis [38]. p53, as a transcription factor, can stimulate the expression of pro-apoptotic target genes, such as PUMA, Bax, and Bid [39]. Moreover, p53 can exert a direct pro-apoptotic function in the mitochondria, thereby activating the mitochondrial apoptotic pathway, which is recently known as the circuitry of p53 death signaling [40]. Death stimulus can induce the translocation of p53 to the mitochondria and the formation of a complex with Bcl-xl and Bcl2, in which the pro-apoptotic BH-3-only proteins, such as PUMA, are released, resulting in mitochondrial membrane permeabilization to release a host of apoptotic activators [41]. In the cytosol, Bcl-2/Bcl-xl protein can sequester Bax protein to inhibit its pro-apoptotic function and translocation to the mitochondria [42]. Our study also demonstrated that quercetin increased DOX-induced PUMA expression, Bax translocation, p53 protein expression, and transcriptional activity as Bcl-xl expression is suppressed. The overexpression of Bcl-xl and pifithrin-α can diminish cell death induced by the co-treatment of DOX and quercetin in hepatoma cells. *In vivo* experiments also demonstrated that p53 expression increased and Bcl-xl expression decreased after the co-treatment with a subsequent decrease in Ki67expression. Tumor sizes were also significantly reduced after quercetin and DOX treatment.

DOX induces cardiotoxicity, nephrotoxicity, and hepatotoxicity. Previous study showed that quercetin can prevent epirubicin-induced acute hepatotoxicity in normal rats [43]. We also confirmed that quercetin decreased DOX-induced acute hepatotoxicity in normal mice. Our data demonstrated that acute administration of DOX increased serum indices of liver function, including ALT and ALP. This increase in ALT and ALP is attributed to the hepatocellular damage and decreased liver functions [44]. Interestingly, quercetin partially reversed DOX-induced increase in serum ALT and ALP. Acute and subacute histopathological changes in DOX-treated livers were also reversed by quercetin administration. The effect of quercetin on DOX-treated human cancer xenografts in mice has not been assessed and mice are routinely used to evaluate the efficacy of drugs in preclinical studies: thus, we investigated the effect of quercetin on DOX-induced chronic hepatic damages in human cancer-xenografted mice. Histopathological changes in DOX-treated livers were also reversed by quercetin administration. Thus, quercetin exhibits a protective effect against DOX-induced acute and chronic liver damage.

Oxidative stress is associated with DOX-induced cell injury and DNA damage. DOX induces hepatic dysfunction by changing the levels of superoxide dismutase, catalase, and glutathione (GSH) enzymes, which are found in the antioxidant enzyme system, in liver tissues [45]. Quercetin also exhibits a protective effect against drug toxicity, which induces oxidative stress. For example, quercetin prevents epirubicin-induced acute oxidative stress toxicity in rat liver cells and mitochondria [43]. Quercetin also increases GSH levels in rats under long-term alcohol consumption-induced oxidative stress [46]. Studies have also shown that quercetin increases the mRNA expression of hepatic enzymes (Gst and Akr) involved in drug metabolism in an isoenzyme-specific manner [47]. Furthermore, studies have shown that acute DOX toxicity alters cytochrome P450 expression in rat liver [48] and quercetin can increase the P-450 reductase activity in human organs [49], suggesting that the presence of quercetin in diet may increase P-450 reductase activity during DOX therapy. Quercetin also suppresses the expression of the pro-apoptotic Bax gene and enhances the anti-apoptotic Bcl-2 gene in normal cells under oxidative stress [50], [51]. Therefore, quercetin can sensitize apoptosis in hepatoma cells without increasing the effect on DOX-induced normal liver cell apoptosis. Further mechanistic studies are needed to verify the relationship between quercetin and antioxidant defense systems in DOX-treated cells and animals.

In summary, the present findings demonstrate that quercetin effectively enhances the toxic effects of DOX in liver cancer cells and hepatoma xenografts. The pro-apoptotic activity of quercetin in DOX-treated liver cancer cells is mediated by p53 accumulation and activation, followed by the mitochondrial apoptotic pathway activation, thereby resulting in the cleavage of pro-caspases that leads to apoptosis (Fig. 6C). However, quercetin reduces this damage in normal liver cells *in vitr*o and *in vivo*. Our results indicate that the combined treatment of quercetin and DOX may be beneficial against human liver cancer, since quercetin can reduce the hepatotoxicity of DOX in normal liver cells.

## Materials and Methods

### Reagents and chemicals

Doxirubicin, quercetin, Hoechst 33258, pifithrin-α, and MTT were from Sigma (St. Louis, MO). JC-1 was from Molecular Probes (Eugene, OR). The caspase inhibitor zVAD-FMK was from R&D Systems Products (Minneapolis, MN). DMSO was from Invitrogen-Life Technologies (Merelbeke, Belgium).

### Cell lines and cell culture

Human hepatoma cell lines SMMC7721, QGY7701 and normal liver cell line L-02 were purchased from Cell Bank (Chinese Acadamy of Sciences). Cells were cultured in RPMI-1640 supplemented with 10% fetal bovine serum and penicillin-streptomycin antibiotics (GibcoBRL, Grand Island, NY). Cells in culture flasks were placed in an incubator with 5% CO_2_ at 37°*C*.

### Cytotoxicity Assay

The cells were triplicate cultured in 96-well plates at a density of 5×10^3^ cells/well overnight and incubated with quercetin (doses: 0 to 150 μM) containing complete RPMI-1640 medium for 48 h. After drug treatment, the medium was replaced with 0.5 mg/ml of MTT in complete medium for 4 h. The surviving cells converted MTT to formazan that generates a blue-purple color when dissolved in DMSO that was measured at 570 nm using a model ELX800 Microplate Reader (Bio-Tek Instruments Inc., Highland Park, USA). The relative percentage of cell survival was calculated by dividing the absorbance of treated cells by the control in each experiment.

### Apoptosis evaluation

Multiple methods were used to analyze apoptotic rates. The first was Hoechst 33258 staining. After drug treatment, the cells were washed with isotonic PBS (pH 7.4) and then fixed in 4% paraformaldehyde solution in PBS for 1 h at 37°*C*. The nuclei were stained with 2 μg/ml Hoechst-33258 for 10 minutes and the apoptotic cells were counted using a fluorescence microscope (Zeiss, Germany).

The second was propidium iodide (PI) staining. 1x10^6^ cells were harvested and washed in PBS, then fixed in 70% ethanol for 24 h at 4°*C*. After centrifugation, the cell pellets were treated with 4 μg/ml PI solution containing 100 μg/ml RNase and 1% Triton X-100 for 30 min. Subsequently, the samples were analyzed in a FACScalibur system (Becton Dickinson, Mountain View, CA). Statistical analysis was performed by Modfit software.

The third used Annexin V- FITC/PI kit (PharMingen). Staining was performed according to the manufacturer's instructions. Flow cytometry was conducted on a FACScalibur system and the results were analyzed by CellQuest software ((Becton Dickinson, Mountain View, CA).

### Caspsase-3 enzyme activity

After treatment, cells were washed with PBS and lysed with cell lysis buffer provided with caspase-3 assay kit (Sigma). Samples were incubated on ice for 10 min and centrifuged in a microcentrifuge at 12,000 g for 5 min at 4°*C*. to precipitate the cellular debris. The caspase-3 activity in the supernatant was measured in a spectrophotometer, using DEVD-p-nitroanilide as a substrate, according to the manufacturer's instructions provided with the assay kit. Triplicate independent experiments were performed.

### Preparation of mitochondrial and cytosol fractions

Cells were washed twice with ice-cold PBS, pH 7.2 and resuspended in extraction buffer (∼500 μl) containing 20 mM HEPESKOH (pH 7.0), 10 mM KCl, 1 mM NaEGTA, 2 mM MgCl2, 1 mM EDTA, 1 mM DTT, 250 mM sucrose, 1 mM PMSF and protease inhibitors cocktail. Lysate was incubated 30 min on ice and then homogenized using a glass dounce (30 strokes). Nuclei were removed by centrifugation at 1,000 g for 10 minutes at 4°C. Supernatant was additionally centrifuged for 20 minutes at 11,000 g and the resulting supernatant (cytosolic fraction) and pellet (mitochondrial fraction) were collected separately and used for Western blotting.

### Western blot analysis

Cells were harvested as described above after drug treatment. Cells were lysed in a sample buffer (Beyond Biotech, China), followed by sonication and heat denaturation. Protein contents were quantified using the Bradford reagent. The protein (40 mg) was applied to a 12% SDS-polyacrylamide gel, transferred to a nitrocellulose membrane. Blots were incubated overnight at 4°C, separately, with the primary antibodies: anti-Bid (1∶1000), Bcl-2 (1∶1000), Bcl-xl (1∶1000), Bax (1∶1000), PUMA (1∶1000), Cytochrome *c* (*1∶500*), anti-caspase-3 (1∶1000), caspase-8 (1∶1000), and caspase-9 (1∶1000)(Cell Signaling, Beverly, MA); anti-p53(1∶2000), PARP (1∶1000)(Santa Cruz Biotechnology, Santa Cruz, CA); anti-β-actin (1∶2000)(Sigma). Blots were incubated for 1 h at room temperature with the appropriate peroxidase-conjugated secondary antibody: Anti-rabbit or anti-mouse IgG horseradish peroxidase (1∶1000) (Zymed Laboratories, Inc., South San Francisco, CA). Visualization was performed with a Molecular Imager FX (Bio-Rad Laboratories, CA) using Kodak ID imaging densitometry analysis software on a Macintosh personal computer.

### Transfection experiments

Stable transfection of SMMC7721 hepatoma cells were performed with plasmid pSFFV-Neo or pSFFV-Bcl-xl (kindly provided by Dr. Steven Grant, Medical college of Virginia, USA.) using lipofectamine transfection reagent (Invitrogen) and cultured in 0.5 mg/ml G418 according to the manufacturer's instructions.

### Luciferase assay

Cells (1×10^5^) suspended in 1 ml of complete medium were seeded into each well of a 12-well plate. After incubating at 37°C for 24 h, cells were transiently transfected by 1 μg of p53-luciferase reporter plasmid and 1 μg of β-gal plasmid in the medium without FBS and antibiotics using the lipofectamine transfection reagent (Invitrogen). The cells were incubated for 4 h and complete medium was added for16 h. The cells were starved for another 16 h in 2% FBS medium, followed by exposure to different treatments. The luciferase activity was determined using the luciferase assay system with reporter lysis buffer from Promega (Madison, WI). Briefly, the cells were harvested by scraping in 200 μl of reporter lysis buffer into a 1.5 ml microcentrifuge tube, vortexed for 15 s, and centrifuged at 12,000 rpm for 30 s, and then the supernatant cell lysates were collected. The luciferase activity was measured with 60 μl of cell lysate and 60 μl of substrate, using a Monolight luminometer. The results are expressed as the relative p53 activity compared with controls after normalizing for β-galactosidase activity and protein concentration.

### Detection of mitochondrial membrane potential

Cells were harvested as described above after drug treatment. 1×10^6^ cells were resuspended after trypsinization in 1ml of medium and incubated with 5 μg/ml of JC-1 for 10 min at 37°C before flow analysis. Red fluorescence emissions were analyzed by CellQuest software in a FACScalibur system (Becton Dickinson, Mountain View, CA).

### Ethics statement and animal treatment protocols

This study was performed in strict accordance with the recommendations in the Guide for the Care and Use of Laboratory Animals of Zhejiang University. The protocol was approved by the Committee on the Ethics of Animal Experiments of Sir Run Run Shaw Hospital (Permit Number: 20110704). Because the daily intake of quercetin in the human diet has been estimated to be in the range of 2–40 mg, we selected 100 mg/kg mice weight of quercetin in this study.

### Acute doxorubicin hepatotoxicity assay in mice

The influence of quercetin on Dox-induced hepatic toxicity was studied in C57BL/6 mice. 6-7 weeks old male C57BL/6 mice were housed in air conditioned, light controlled animal facilities and randomly divided into four groups (n = 6 per group): control (group 1), Dox-treated (group 2), quercetin-treated (group 3), Dox and quercetin cotreated (group 4). Before Dox application, in group 3 and group 4, a treatment with quercetin (100 mg/kg/day, p.o.) was started for 4 consecutive days to maintain the quercetin concentration in mice plasma. The other two groups received saline. Dox (20 mg/kg) was administered intraperitoneally at day five in group 2 and 4. Five days after Dox injection, mice were sacrificed. A blood sample of each animal was collected into a dry centrifuge tube. Serum was separated by centrifugation at 3000 r.p.m/15 minutes and used to determine alanine aminotransferase (ALT) and aspartate aminotransferase (AST) by enzyme assay kits (ALT/AST assay kit; DiaSys Diagnostic Systems (Shanghai) Co., Ltd, China) according to the manufacturer's instructions. This measurement was done with a 7020 chemistry analyzer (Hitachi, Japan). For determination of the histopathological changes, livers of mice from different groups were removed by dissection.

### Human tumor xenograft experiment

4–6 weeks old female nude athymic BALB/c nu/nu mice were housed and maintained in laminar flow cabinets under specific pathogen-free conditions. *In vitro* cultured human hepatic cancer SMMC7721 cells (5×10^6^ in 200 μl volume) were injected *s.c*. into the right supra scapula region of mice. Tumor volume was estimated by using the formula volume  =  lengthxwidth^2^/2. When tumors grew to an average volume of 75 mm^3^, mice were randomly divided into four groups (five mice per group) and treated i.p. with 100 mg/kg quercetin three times a week (Group 1), 4 mg/kg DOX once a week (Group 2), a combination of 4 mg/kg DOX and 100 mg/kg quercetin (Group 3) or vehicle control injected with the same volume of saline (Group 4) for three weeks. The tumor volumes were determined by caliper measurement twice a week. When control mice started to succumb to their tumors, the mice in all treatment groups were euthanized and the tumors were weighed for treatment efficacy. Tumor tissue and liver samples from mice were isolated for histopathological evaluation.

### Histopathology

Tissue samples were fixed in 10% formol saline for 24 hours and embedded in paraffin, cut in 4 mm sections, stained with Harris Hematoxylin & Eosin and evaluated for any structural changes under a bright field microscope. Standard immunoperoxidase procedures were used to visualize Ki67, p53, and Bcl-xl in tumor samples. Briefly, sections were deparaffinized, blocked with goat serum, followed by incubation with anti- Ki67 (1∶150), anti-p53 (1∶150) or anti-Bcl-xl (1∶100) overnight at 4°C. After incubation with horseradish peroxidase linked secondary antibody for 30 min, the sections were counterstained with Mayer's hematoxylin.

### Statistical analysis

Results were subjected to computer-assisted statistical analysis using the ANOVA one-way analysis of variance and the Tukey-Kramer single-step multiple comparison procedure as a post test. Differences of P<0.05 were considered significant.
